# Resting-state fMRI analysis of functional connectivity and temporal dynamics differences between cocaine users and healthy controls

**DOI:** 10.1016/j.ynirp.2025.100304

**Published:** 2025-12-17

**Authors:** Sravani Varanasi, Tianye Zhai, Hong Gu, Betty Jo Salmeron, Yihong Yang, Fow-Sen Choa

**Affiliations:** a Department of Computer Science and Electrical Engineering, University of Maryland Baltimore County, Baltimore, MD, 21250, USA; b Neuroimaging Research Branch, Intramural Research Program, National Institute on Drug Abuse, National Institutes of Health, Bethesda, MD, 28092, USA

## Abstract

Understanding alterations in functional connectivity among individuals with substance use disorder (SUD) is critical for elucidating the neural mechanisms underlying addiction. In this study, we applied Energy Landscape Analysis (ELA), an energy-based machine learning method, to examine whole-brain functional connectivity differences between SUD patients and healthy controls (HCs). A key methodological challenge in ELA lies in the selection of appropriate Regions of Interest (ROIs) from comprehensive brain atlases. To address this, we employed seed-based connectivity analysis to identify task-relevant ROIs, thereby overcoming the limitation of focusing on a restricted subset of regions. The dataset comprised 53 cocaine users (CUs) and 52 age- and sex-matched HCs, with functional MRI data preprocessed using the CONN toolbox. ROI-to-ROI seed-based connectivity was computed through first- and second-level analyses. ELA revealed that HCs exhibited stronger positive connectivity between cerebellar and visual regions, whereas CUs showed stronger positive connectivity between the cerebellum and the inferior temporal gyrus (temporooccipital part; toITG). Seven low-energy connectivity states were identified that differentiated the two groups. In these states, the cerebellum and toITG demonstrated antagonistic activation patterns, while the cerebellum and visual cortex co-activated in HCs. Temporal dynamics analyses further indicated that HCs visited these low-energy states more frequently, driven by shorter dwell times but higher transition rates. These findings suggest that cocaine addiction may reflect a weakening of adaptive, protective (“guardian”) connectivity patterns, rather than an increased propensity to remain in maladaptive connectivity states. Collectively, these results highlight key network-level distinctions between HCs and CUs and offer new insights into the neurobiological mechanisms of cocaine addiction.

## Highlights

• Energy Landscape Analysis (ELA) explored whole-brain ROI functional connectivity differences between Cocaine users and healthy controls using CONN toolbox for ROI selection.
• Cocaine users showed reduced connectivity between the superior temporal gyrus, basal ganglia network, and visual network and cerebellum.
• Cocaine users showed stronger connectivity between the inferior temporal gyrus (toITG) and the cerebellum.
• Distinctive states showed antagonistic connectivity between the cerebellum and toITG, and cooperative connectivity between the cerebellum and primary visual network.
• HCs visited low-energy states more often, suggesting functional brain state differences are based on occurence frewuency and not dwell times.

## 1 Introduction

Computational analysis of functional magnetic resonance imaging (fMRI) data has become an indispensable tool for studying neural mechanisms underlying various clinical conditions, including substance use disorders such as cocaine addiction. Resting-state functional magnetic resonance imaging (rs-fMRI) has been central to revealing systems-level abnormalities, reporting disrupted functional connectivity within and between the default mode network (DMN), salience network (SN), executive control network (ECN), mesocorticolimbic circuits, and thalamostriatal pathways in individuals with cocaine use compared with healthy controls (HCs). (Xu et al., 2024; Geng et al., 2017; Liang et al., 2015; Ray et al., 2016). The ability to detect subtle alterations in how brain regions coordinate activity—particularly at rest—offers profound insights into the neurobiology of drug dependence, beyond what can be revealed through task-based imaging alone (Yang and Lewis, 2021; Yuste and Fairhall, 2015).

One crucial advance in this field is the study of temporal dynamics using resting-state fMRI data (Yuste and Fairhall, 2015). Beyond static connectivity, the human brain at rest exhibits rich temporal dynamics, transitioning among recurrent connectivity “states” with measurable properties such as visitation frequency, dwell time, and transition probabilities. Dynamic analyses capture these moment-to-moment reconfigurations and have proven sensitive to aging, cognition, and neuropsychiatric disease, motivating their application to addiction. However, common frameworks (e.g., sliding-window correlation, co-activation patterns, and point-process/state clustering) often assume linear dynamics or rely on heuristic choices (window sizes, cluster numbers) that can obscure nonlinear, multistable behavior (Valsasina et al., 2019; Maltbie et al., 2022; Palmer et al., 2022).

Energy Landscape Analysis (ELA) offers such a principled perspective by linking observed brain-state configurations to a probabilistic “landscape” whose local minima represent attractor states and whose barriers govern transition likelihoods. Typically instantiated via pairwise maximum entropy modeling (pMEM), ELA estimates the relative “energy” of multivariate activity/connectivity patterns, allowing inference about which states are most stable, how frequently they are visited, and how transitions are organized via saddle (hub) states. ELA has been validated across healthy and clinical cohorts and provides reliability advantages when carefully implemented, positioning it as a compelling tool for interrogating the nonlinear dynamics of addiction-relevant networks (Ezaki et al., 2017; Kang et al., 2017, 2019; Khanra et al., 2024; Varanasi et al., 2023). Recent applications of ELA to rs-fMRI demonstrate that disease processes can reshape attractor structure—altering the repertoire, stability, and transition architecture of brain states. Studies in neurodegeneration, for example, report condition-specific differences in the size and occupancy of major states and in the ease of switching between them, highlighting ELA's sensitivity to clinically meaningful alterations in network dynamics. Methodological work further shows that surrogate-data tests and robust pipelines can quantify the specificity of inferred landscapes, strengthening confidence in ELA-derived biomarkers (Xing et al., 2024; Jing et al., 2025; Ishida et al., 2024).

Previous fMRI research in cocaine users has repeatedly demonstrated significant differences from healthy controls, with reports of altered activation in regions such as the insula, parietal cortex, striatum, and frontal gyri (Moeller et al., 2010; Terenzi et al., 2024; Dang et al., 2022). Additionally, abnormal effective and functional connectivity within reward circuits—including the mesocorticolimbic system—has been linked to core symptoms of addiction and impaired cognitive control (Ray et al., 2016). While studies using dynamic causal modeling and other methods have contributed to this understanding, ELA offers a uniquely data-driven, temporally resolved perspective that goes beyond static group averages (Khanra et al., 2024; Hosaka et al., 2025).

The present study leverages ELA on rs-fMRI from cocaine users and matched healthy controls to calculate the functional connectivity differences and temporal dynamics between the different ROIs. By integrating an established addiction neuroscience literature with a nonlinear, probabilistic account of brain dynamics, our work aims to (a) identify disease-relevant “most visited” or lowest-energy connectivity states, and (b) quantify alterations in temporal dynamics (visitation and dwell). The present work is significant because it combines the robust, theory-driven approach of energy landscape analysis with meticulous comparison between cocaine users and healthy controls. By mapping both temporal dynamics and the most frequently visited brain connectivity states, this research advances the field's ability to pinpoint the neurobiological signatures of addiction—and ultimately informs intervention strategies targeting these dynamic patterns.

## 2 Materials and methods

### 2.1 Dataset and preprocessing

In this study, we utilized resting-state fMRI data of 52 CUs and 53 age- and sex-matched HC subjects. The study was approved by the institutional review board of the National Institute on Drug Abuse Intramural Research Program and all participants provided informed consent. The 52 CUs were active, non-treatment seeking individuals, with ages 39.81 ± 6.74 years, while the 52 HC subjects had an age of 38.04 ± 7.59 years.

We employed CONN (Nieto- and Castanon, 2020; Whitfield-Gabrieli and Nieto-Castanon, 2012), a Statistical Parametric Mapping (SPM) based cross-platform software, for the computation, display, and analysis of fMRI data. We used CONN's default preprocessing pipeline which includes Functional realignment and Unwarp, Slice Timing Correction, Outlier Identification, Direct Segmentation and Normalization and Functional Smoothing. It begins with realignment and unwarping, where head motion across scans were corrected, followed by slice-timing correction to adjust for differences in slice acquisition timing within each TR. Outlier scan were identified using Artifact Detection Tools (ART) toolbox, flagging those with framewise displacement over 0.9 mm or signal intensity more than 3 standard deviations from the mean. Next, the structural (T1-weighted) image undergoes segmentation into gray matter, white matter, and CSF, and is normalized to standard Montreal Neurological Institute (MNI) space, after which the same transformation has been applied to the functional images for spatial alignment across the subjects. The normalized functional data are then smoothed using an 8 mm full width half maximum (FWHM) Gaussian kernel to enhance signal-to-noise ratio and accommodate inter-subject variability.

After preprocessing, we conducted Quality Assurance (QA) checks to examine the output of some preprocessing steps. This was followed by the denoising step, which is crucial for cleaning noise from resting-state fMRI data. Once denoising was completed, we proceeded with first level and second level analyses. The second-level analysis (also known as group-level analysis) is the stage where statistical inferences are made across subjects, allowing us to identify patterns of functional connectivity differences or similarities between the two groups of subjects. CONN employs the General Linear Model (GLM) for all second-level analyses of functional connectivity data.

### 2.2 ROI-to-ROI connectivity (RRC)

During the first and second level analysis in the CONN we performed the ROI-to-ROI connectivity (RRC) analysis, which describe the connectivity between every pair of ROIs within a predefined set of regions. RRC matrices represent the level of functional connectivity between pairs of ROIs. Each element in an RRC matrix is defined as the Fisher-transformed bivariate correlation coefficient between the BOLD time series of a pair of ROIs.

The Atlas used in this study is the default atlas from the toolbox which is Harvard-Oxford atlas. This atlas has a total of 164 ROIs including the 132 atlas ROIs and 32 network ROIs. For the RRC analysis we have used the 132 atlas ROIs in this study, these 132 ROIs belong to 22 clusters/networks as per the CONNs default ROI clustering and ordering procedures (Sorensen, 1948; Bar-Joseph et al., 2001). The 132 ROIs and their corresponding network affiliation as per the CONN Harvard-Oxford atlas are shown in Table S1 of the Supplementary material.

In the second-level ROI-to-ROI analysis, we generated a connectome ring, as illustrated in Fig. 2. This analysis utilized a between-subjects contrast, specifically comparing CUs to HCs, with a contrast vector of (Xu et al., 2024). The connectome ring was generated using standard settings for cluster-based inferences, specifically Functional Network Connectivity (FNC) (Nieto- and Castanon, 2020). In the FNC approach, clusters are identified through a data-driven hierarchical clustering method known as complete-linkage clustering (Whitfield-Gabrieli and Nieto-Castanon, 2012), which is based on anatomical proximity and functional similarity metrics between ROIs. The connectome ring was derived by selecting atlas ROIs from the default atlas provided by the CONN toolbox, applying a connection threshold of p < 0.05 for uncorrected p-values and a cluster threshold of p < 0.05 for cluster-level p-FDR corrections.

### 2.3 Maximum entropy model

To analyze this dataset, the concept of energy landscape analysis described in the previous studies (Ezaki et al., 2017; Kang et al., 2017, 2019; Khanra et al., 2024; Varanasi et al., 2023) has been used. This concept is based on the pairwise Maximum Entropy Model (MEM) estimation approach, and the algorithm used for the estimation process is Maximum likelihood estimation (MLE). Firstly the ROIs (i=1,…,N) are selected, the number of ROIs can be represented as N, and for every N ROIs the number of possible connectivity states can be given as $2^{N}$. After the ROIs are identified, the fMRI signal at each ROI is binarized into 1 being active or −1 being inactive by thresholding the signal. The threshold is arbitrary and is set to the time average of the data at each ROI.

Next, the relative frequency with which each activity pattern is visited is calculated which is then fitted to the Boltzmann distribution given by

$$P\left(\sigma |h,J\right)=\frac{\exp\left[-E\left(\sigma |h,J\right)\right]}{\sum_{\sigma^{\prime }}\exp{\left[-E\left(\sigma^{\prime }|h,J\right)\right]}^{\prime }}$$

where,

$$E\left(\sigma |h,J\right)=-\sum_{i=1}^{N}h_{i}\sigma_{i}-\frac{1}{2}\sum_{i=1}^{N}\sum_{\begin{array}{c} j=1\\ j\neq i \end{array}}^{N}J_{ij}\sigma_{i}\sigma_{j}$$

is the energy, $h=\left\{h_{i}\right\}$ and $J=\left\{J_{ij}\right\}\left(i,j=1,\ldots ,N\right)$ are the parameters of the model, $\sigma =\left\{\sigma_{i}\left(1\right),.\;.\;.,\sigma_{i}\;\left(t_{\max}\right)\right\}$ is the binarized signal representing the brain activity for each ROI and $t_{\max}$ is the length of the data (Ezaki et al., 2017). Thus, the Boltzmann distribution equation proposes that the activity pattern with high energy does not have a high probability to show up and vice versa.

The model parameters h and J are estimated using Maximum Likelihood Estimation (MLE) as shown below

$$\left(h,J\right)=\arg\max_{h,J}L\left(h,J\right)\text{,}$$

where L(h,J) is the likelihood given by

$$L\left(h,J\right)=\prod_{t=1}^{t_{\max}}P\left(\sigma \left(t\right)|\;h,J\right)\text{.}$$

The MLE becomes increasingly computationally demanding as the number of ROIs (N) grows, due to the exponential increase in the number of possible activity patterns at each step (2^N^). Consequently, the inclusion of a large number of ROIs in the energy landscape analysis (ELA) is not feasible, making the selection of ROIs a critical methodological consideration.

### 2.4 ROI selection

The ELA requires us to select the ROIs for the Maximum Entropy Model and to do that we used CONN's RRC analysis results. The RRC analysis using CONN resulted in twenty-one ROIs which are clustered into six networks as per the Harvard-Oxford atlas clustering (Table 1). We have added a seventh network ROI which is Thalamus, so that we can analyze the cortico-basal ganglia-thalamic-cortical loop (for future studies). So, we have used a total of seven ROIs for the ELA namely, Superior Temporal Gyrus (STG), Inferior Temporal Gyrus, temporooccipital part (toITG), Visual primary (Vis.Primary), Auditory (AUD), Cerebellum, Basal Ganglia (BSL) and Thalamus (THL).

**Table 1:** ROIs from connectome ring in Fig. 2 and corresponding network affiliations.

ROI name	ROI abbreviation	Network affiliation
**Superior Temporal Gyrus, anterior division**	aSTG	Superior Temporal Gyrus
**Superior Temporal Gyrus, posterior division**	pSTG	
**Inferior Temporal Gyrus, temporooccipital part**	toITG	toITG
**Intracalcarine Cortex**	ICC	Visual. Primary
**Cuneal Cortex**	Cuneal	
**Lingual Gyrus**	LG	
**Supracalcarine Cortex**	SCC	
**Parietal Operculum Cortex**	PO	Auditory
**Planum Polare**	PP	
**Heschl's Gyrus**	HG	
**Planum Temporale**	PT	
**vermis 8**	Ver8	Cerebellum
**vermis 7**	Ver7	
**vermis 6**	Ver6	
**vermis 4 5**	Ver45	
**Cerebelum 4 5**	Cereb45	
**Cerebelum 6**	Cereb6	
**vermis 3**	Ver3	
**Caudate**	Caudate	Basal Ganglia
**Putamen**	Putamen	
**Accumbens**	Accumbens	

### 2.5 Energy landscape analysis

The pairwise MEM method is applied to all the selected ROIs STG, toITG, Vis.Primary, AUD, Cerebellum, BSL and THL separately. After the Pairwise MEM is applied, the energy values at each activity state are computed using MEM model. Next, the Dijkstra algorithm is applied to the energy values to plot the disconnectivity graph, this algorithm helps to organize the disconnectivity graph and find out the relationship between the neighboring nodes. The local minimums are then plotted as an activity pattern which allows us to visualize the connectivity between the different ROIs.

Below is an example of the disconnectivity graph and activity map of one of the healthy controls.

**Fig. 1:**
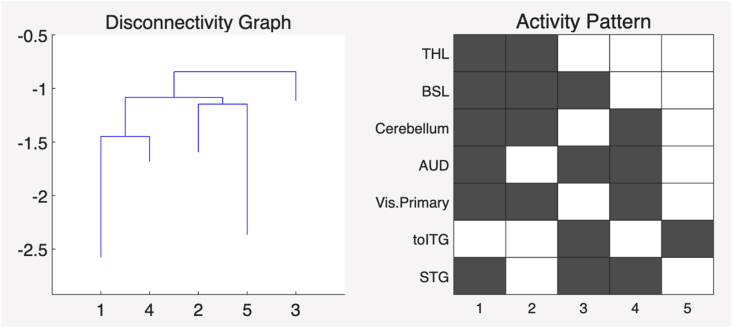
Disconnectivity graph and activity pattern of one of the healthy controls.

The disconnectivity graph (in Fig. 1) illustrates the relationships among local minima, where each leaf corresponds to a local minimum derived from the activity patterns. The branching structure reflects the energy differences between these local minima, with each minimum representing a distinct connectivity state characterized by its specific activity pattern. The black in the activity pattern represents the ROI being inactive (or 0) and white being active (or 1). In the disconnectivity graph, the y-axis represents the energy of the local minima, and the x-axis represents their corresponding state numbers from the activity pattern. The energy of each local minimum is inversely related to the likelihood of visiting that particular connectivity state. Which means, the leaf positioned deeper in the disconnectivity graph (indicating lower energy levels) correspond to states with a higher probability of being visited.

After obtaining the energy values for all the CUs and HCs through ELA, the energy values were used to calculate the p-values. A statistical method called Bonferroni correction was used to control the risk of false positives or Type I errors because of multiple hypothesis tests performed. So, to satisfy the Bonferroni correction the resulted p-value should be less than p-value, 0.05, divided by the total number of possible connectivity states (in this case $2^{7}$ or 128).

### 2.6 Temporal dynamics

Temporal dynamics in ELA refer to how the brain's functional connectivity patterns change over time that is, how the brain transitions between different “states” of network activity. ELA models brain activity (often derived from fMRI data) as a dynamic system that can occupy multiple stable or metastable “states.” Each state corresponds to a specific pattern of functional connectivity among different brain regions or ROIs. The MEM model is used to calculate the energy and the probability values for each functional connectivity state. The energy represents the stability or likelihood of a state, low energy state means stable/frequently visited states and high energy means unstable/rarely visited states. Using the information on the local minimums an energy landscape can be plotted where temporal dynamics capture how the brain moves through this landscape over time — how often it visits each state, how long it stays there, and how it transitions between states. The dynamics of the

Activity patterns are illustrated as the motion of a ‘ball’ on the energy landscape. This gives us the key metrics which include the visit count: how frequently each state is visited during the scan, dwell time (or persistence): how long the system stays in each state before switching and state transitions/switching rate: the overall flexibility or stability of the system.

## 3 Results

**Fig. 2:**
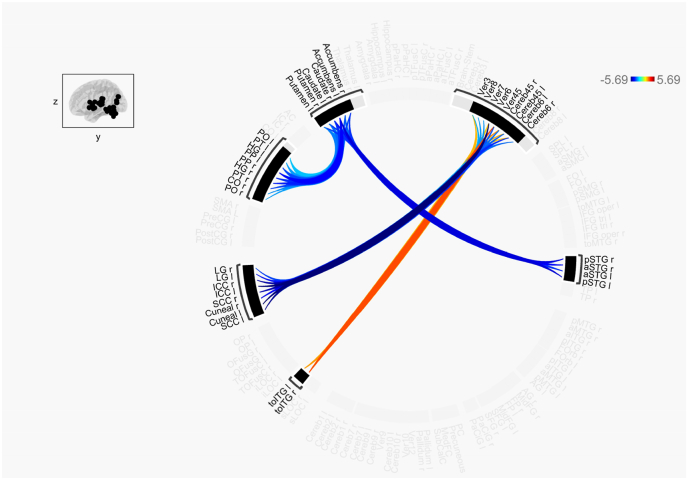
ROI-ROI connectivity, for the difference CUs minus HCs. The connection threshold was set to p < 0.05 for p-uncorrected and a cluster threshold at p < 0.05 for cluster level p-FDR corrected.

The CONN toolbox's ROI-ROI connectivity analysis allowed us to look at the functional connectivity differences between CUs and HCs, which is illustrated in Fig. 2. Four clusters of connectivities can be seen in the connectome ring. The ROIs from these clusters belong to different networks per the CONN default atlas as shown in Table 1.

When comparing two groups using the connectome ring, with the between-subject contrast of (Xu et al., 2024) (indicating the difference CUs > HCs), the effect sizes from the resulting *t*-test reflect the differences in connectivity between the two groups. Specifically, red/positive values indicate stronger connectivity in CUs compared to HCs, while blue/negative values indicate stronger connectivity in HCs compared to CUs. Based on this interpretation, as illustrated in Fig. 2, CUs exhibit stronger connectivity between the toITG and ROIs within the Cerebellum. In contrast, HCs display stronger connectivity between ROIs within the STG and those belonging to the BSL network, as well as stronger connectivity between ROIs within the Visual primary network and the Cerebellum.

Using the ROIs from Table 1 and an additional ROI which is Thalamus, Energy landscape analysis was yielding energy values for all possible connectivity states. These energy values of CUs and HC subjects connectivity states were then compared to calculate p-values. We identified a total of 48 connectivity states with p-values less than 0.05. For the seven ROIs there were a total of $2^{7}$ or 128 possible connectivity states resulted from ELA. After applying Bonferroni correction, eight connectivity states remained significantly different, meaning having a p-values less than 0.05/128.

**Fig. 3:**
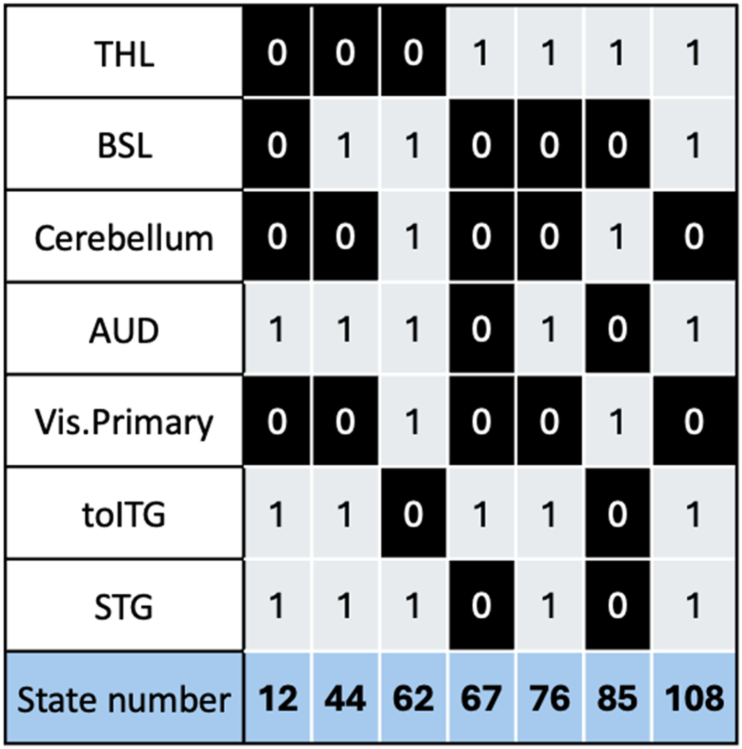
Connectivity states that satisfy the Bonferroni correction by comparing the energy values of CUs an HCs resulted from ELA.

In the ELA procedure, when a particular local minimum has lower energy, the neighboring states may also have lower energy, the neighboring states are the ones which were transitioned in the processes of finding the actual local minima. But it is not necessary that the neighboring state will also become a local minimum. So even though we have a state which was statistically significant but since it is not a local minimum resulted from the ELA calculations, we have omitted that state from further analysis, meaning the omitted state could be a neighboring state to one of the local minima states. Fig. 3 highlights the connectivity states that satisfy the Bonferroni correction and are local minima states with low energy values.

The ELA originally is binarized into -1's and 1's, where −1 is inactive state and 1 is an active state. But in this work for easy visualization −1 (inactive state) are represented as 0's in the activity patterns (Fig. 3). So, in Fig. 3, the 0s (black) and 1s (white) indicate whether an ROI is in an inactive or active state, respectively. The state numbers used in Fig. 3 are binary to decimal conversion of the connectivity pattern (vertically), plus one, which are used to label corresponding to specific connectivity states.

**Fig. 4:**
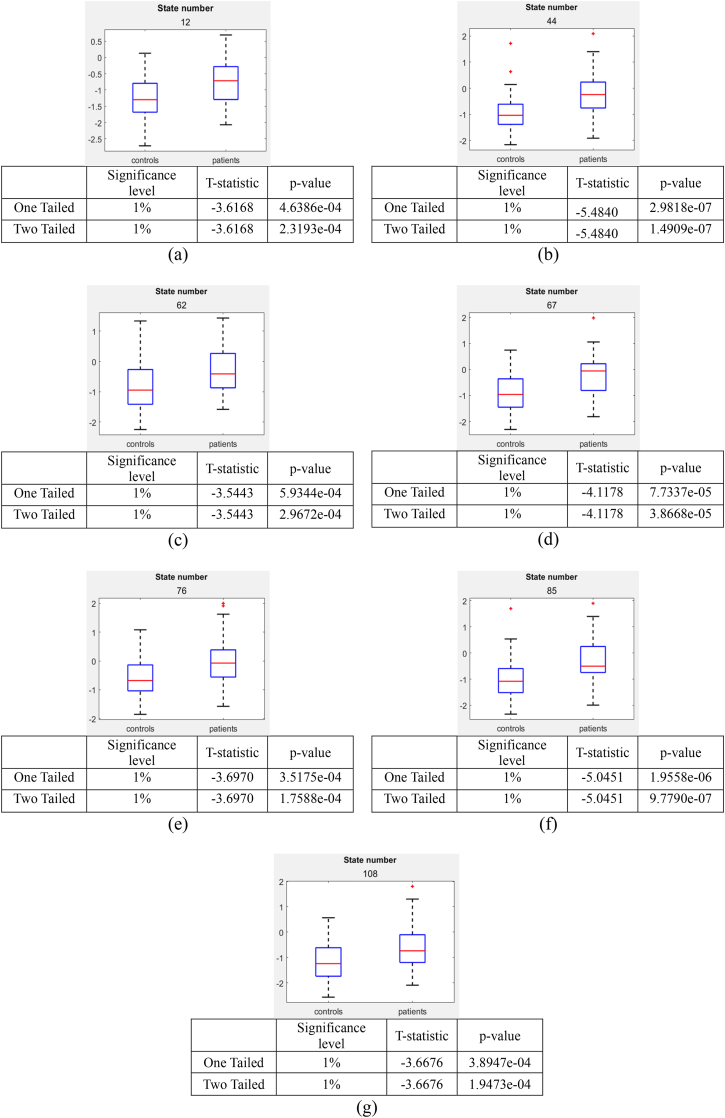
Two−sample t−test was performed on the connectivity states that satisfy the Bonferroni correction from Fig. 3, and are considered potential connectivity signatures (a) Box plot and *t*-test results for the connectivity state 12, (b) Box plot and *t*-test results for the connectivity state 44, (c) Box plot and *t*-test results for the connectivity state 62, (d) Box plot and *t*-test results for the connectivity state 67, (e) Box plot and *t*-test results for the connectivity state 76, (f) Box plot and *t*-test results for the connectivity state 85 and (g) Box plot and *t*-test results for the connectivity state 108.

The box-and-whisker plots were generated for the Bonferroni corrected connectivity states (from Fig. 3) and are displayed in Fig. 4 along with the two-sample *t*-test results. The box plots help us to understand which group of subjects have lower energy values. The lower the variance energy value in the box plot the more stable is the connectivity for that subject group.

From Fig. 3, examination of the horizontal connectivity patterns reveals that all connectivity states display a distinctive antagonistic relationship between the cerebellum and the toITG. Specifically, when Cerebellum is active, toITG is inactive, and vice versa. The boxplots in Fig. 4 further demonstrate that healthy controls (HCs) consistently exhibit lower median energy values across all connectivity states, suggesting that this antagonistic interaction between the cerebellum and toITG is more pronounced in HCs. Another notable pattern observed in Fig. 3 is that primary visual cortex and the cerebellum are working together meaning they are active and inactive together. As shown by the boxplots, this connectivity is stronger in HCs compared with CUs.

Additionally, another pair of ROIs, the AUD and STG networks, show strong correlations across all states depicted in Fig. 3. This correlation is more prominent in HCs, suggesting that the AUD and STG networks are more strongly correlated in HCs compared to CUs.

**Fig. 5:**
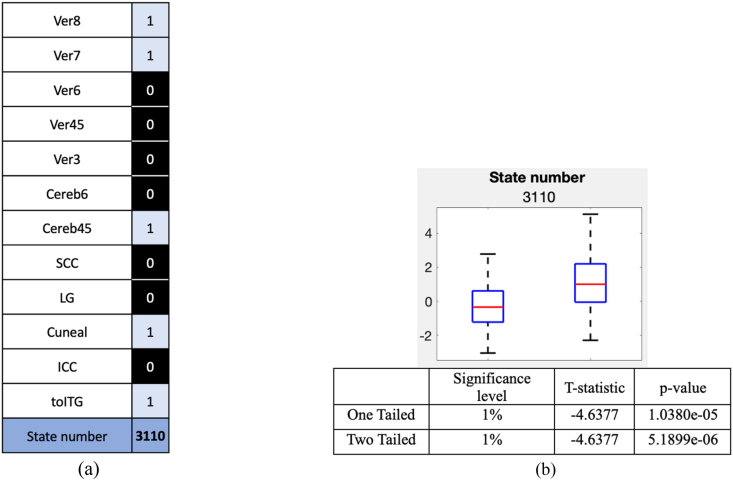
(a) Connectivity states which satisfy Bonferroni correction for the intra network connectivity between Cerebellum, Visual Primary and toITG networks ROIs. (b) Two−sample t−test was performed on the connectivity states that satisfy the Bonferroni correction from Fig. 4(a). Box plot and *t*-test results for that connectivity state are shown.

We further conducted a between-network analysis of the ROIs that showed significant impact based on the previous findings. The networks analyzed include the Cerebellum, toITG, and primary visual areas, totaling 12 ROIs (as listed in Table 1), which resulted in 4096 possible connectivity states after the ELA. Among these, 960 states had p-values below 0.05, and one state met the criteria for Bonferroni correction. The Bonferroni-corrected state and their corresponding boxplots are presented in Fig. 5(a) and (b).

The state in Fig. 5(a) indicates that the ROIs Ver7, Ver8, Cereb45, Cuneal, and toITG function in tandem, exhibiting either collective activation or inactivation. Notably, the cerebellar vermis regions display signs of dysfunction in these states, with connectivity appearing significantly stronger in HCs (observed in boxplots in Fig. 5(b)). This suggests that the connectivity of the cerebellar vermis lobules is disrupted in individuals with long-term cocaine use.

We performed a comprehensive analysis of state transitions across the entire time series for subjects in each group to investigate temporal dynamics and changes in connectivity states. This allowed us to track the frequency of visits to each connectivity state and determine its dwelling time, representing how long a state remains active in a particular connectivity state. Table 2 shows the maximum dwelling times for each Bonferroni corrected state among the CUs and HCs. The dwelling time here represents the amount of time a connectivity state is retained (or transitioned to itself) in the same state in the total scan time.

**Table 2:** Maximum dwelling times for each Bonferroni corrected state among the (i) CUs group and (ii) HCs group.

(i)	
Max dwelling time for each Bonferroni corrected state	
State number	max dwelling time

12	5
44	5
62	3
67	3
76	3
85	4
108	5

(ii)	
Max dwelling time for each Bonferroni corrected state	
State number	max dwelling time

12	4
44	2
62	3
67	3
76	2
85	3
108	3

The dwelling times from Table 2 for the HCs group are not significantly higher compared to those of the patient group. Which means the lower energy Bonferroni corrected states are not a result of the higher dwelling times. To further investigate, we also calculated the number of visits to the Bonferroni-corrected states (Fig. 3) for all CUs versus HCs.

Table 3 summarizes the total number of visits for both HCs (Table 3(i)) and CUs (Table 3(ii)). Interestingly, the HCs group—which exhibits lower median energy levels compared to the CUs (as shown in Fig. 4 boxplots)—had a higher number of visits to the Bonferroni-corrected states.

**Table 3:** The total number of state visit counts, (i) for HCs and, (ii) for CUs.

(i)	
**State number**	**Total visit count**

12	173
44	121
62	137
67	123
76	107
85	150
108	177

(ii)	
**State number**	**Total visit count**

12	132
44	64
62	72
67	77
76	52
85	78
108	102

We also analyzed the state transition data to examine how often a Bonferroni-corrected state transitions to a different state, or how often other states transition into a Bonferroni-corrected state throughout the entire time series. Table 4 presents the transition counts, excluding transitions where the state remains the same (i.e., self-transitions).

**Table 4:** This table shows all the possible state transitions count to and from the Bonferroni corrected states for (i) HCs and (ii) CUs.

(i)		
State number	total state transition from state	total state transition to state

12	117	117
44	81	83
62	98	100
67	89	88
76	77	77
85	104	104
108	112	114

(ii)		
State number	total state transition from state	total state transition to state

12	84	83
44	57	55
62	52	52
67	60	60
76	45	45
85	60	59
108	72	73

Temporal dynamics analysis indicated that the Bonferroni-corrected connectivity states—representing the lowest-energy, most stable configurations—were visited significantly more frequently by HCs than by CUs. These states also exhibited a higher number of transitions in HCs, reflecting greater flexibility in engaging and disengaging from stable network configurations. As shown in Fig. 3, HCs demonstrated lower median energy values across these states, further supporting their enhanced network stability.

Importantly, the mean dwell time within these low-energy states did not differ significantly between groups, suggesting that the observed group differences are driven by visit frequency and transition dynamics rather than by sustained occupancy. Together, these findings suggest a potential mechanism in which stable, low-energy connectivity patterns in HCs may serve as a regulatory or “braking” system. In contrast, the reduced engagement with these states in CUs suggests a disruption in this mechanism, potentially impairing their ability to modulate neural activity effectively, which could contribute to the persistence of addictive behavior.

## 4 Discussion

This study provides novel insights into disrupted large-scale brain network organization in individuals with cocaine use. Using the CONN toolbox's RRC analysis results to find the significant connectivity between the pair of ROIs from the predefined set of ROIs, this allows selection of ROIs for the ELA. With Energy Landscape Analysis (ELA), we demonstrated that cocaine exposure alters both static and dynamic properties of neural communication—particularly involving the cerebellum, inferior temporal gyrus (toITG), and visual cortices. These results extend current addiction models by highlighting how sensory-associative integration and cerebellar modulation may contribute to maladaptive cognitive control and perceptual rigidity.

The antagonistic interaction between the cerebellum and toITG identified in this study likely reflects impaired top-down regulation of associative learning and visual memory processes. This inverse coupling pattern suggests that cocaine usage disrupts the cerebellum's predictive and modulatory role in cortical processing, leading to inefficient coordination of attention and sensory encoding. Conversely, the strong co-activation between cerebellar and visual regions in healthy controls indicates intact cross-modal integration and feedback control. These findings align with prior evidence that cerebellar circuits support cognitive prediction, reinforcement learning, and sensorimotor coordination, all of which are dysregulated in addiction (Pastor and Miquel, 2023).

Alternative explanations for these disrupted connectivity patterns could include chronic exposure to cocaine leading to neuroplastic changes, heightened allostatic load, or compensatory mechanisms that alter how sensory and motor networks interact. The absence of strong visual-cerebellar connectivity in cocaine users may also be influenced by polysubstance abuse, differences in comorbidity profiles, or other unmeasured clinical variables.

Importantly, the observed connectivity abnormalities may not be exclusive to cocaine use. Similar disruptions in cerebellar and visual networks have been observed in individuals with opioid, alcohol, and nicotine dependence, though the spatial extent and direction of these effects differ (Camchong et al., 2011; Janes et al., 2013; Tomasi et al., 2010a). The pronounced cerebellar–ITG antagonism and reduced low-energy state stability in CUs suggest stimulant-specific neuroadaptive processes, possibly driven by dopaminergic toxicity or compensatory neural hyperactivity. Future comparative studies across substance types will help determine whether these connectivity features serve as transdiagnostic addiction markers or distinct signatures of stimulant dependence.

From the intra-network ROIs analysis, a dysfunction between the cerebellar vermis lobules was observed, specifically between Ver7, Ver8 and Ver3, Ver45, Ver6 regions. This type of disrupted connectivity can result in impaired cognitive function, emotional regulation, and motor control, all of which are frequently observed in cocaine users. Emerging studies support the view that cerebellar lobules not only handle motor functions but also have a significant role in non-motor cognitive processes (Lawrenson et al., 2018).

The higher visit count of low-energy, Bonferroni-corrected connectivity states in HCs suggests more efficient dynamic regulation of large-scale brain networks. Frequent transitions into these low-energy connectivity states indicate that HCs can flexibly stabilize neural activity when required—a hallmark of adaptive network control and cognitive resilience (Bassett and Sporns, 2017; Braun et al., 2015). In contrast, CUs exhibited lower visit count and transition frequency among these stable states, implying a disruption in the intrinsic mechanisms that maintain network homeostasis. Such alterations are consistent with prior evidence of dysregulated cerebellar–cortical and fronto-striatal interactions in substance use disorders, where impaired communication between regulatory and reward circuits compromises executive control and behavioral inhibition (Tomasi et al., 2010b). The absence of significant group differences in dwell time further indicates that the impairment in CUs does not arise from prolonged occupation of unstable configurations, but from a reduced capacity to re-enter low-energy states efficiently. Overall, these findings support a model in which cocaine use disrupts the balance between stability and flexibility across functional networks, weakening the brain's regulatory “braking” system essential for maintaining behavioral control.

Clinically, the altered cerebellar–temporal–visual dynamics observed here may contribute to attentional bias, impaired inhibition, and difficulty integrating sensory cues with goal-directed behavior—symptoms commonly seen in CUs. The reduced engagement with low-energy, stable states in CUs points toward impaired neural “braking” capacities—improving these may support better impulse control and treatment outcomes. Future therapeutics might focus on restoring or compensating for disrupted cerebellar–toITG/visual network interactions, either via cognitive interventions, neurofeedback training or targeted brain stimulation techniques. Importantly, identification of these distinct network-level signatures could serve as biomarkers for clinical staging or treatment response in cocaine users.

The question of specificity to cocaine remains important, as certain features—such as disrupted cerebellar vermis connectivity—have been reported in cocaine addiction and in other SUDs, but not consistently across behavioral addictions. The present findings imply that the cerebellum's role in associative memory and reward-driven learning may be particularly sensitive to the effects of chronic cocaine use, in part due to its unique impact on dopamine signaling and neuroplasticity in these regions. Direct comparative research is warranted.

This study's approach and findings should be interpreted considering the broader neuropharmacological literature using fMRI to map substance-induced changes in brain connectivity. The recent connectomics work in stimulant use disorders (Ghazi et al., 2025), provide an important methodological framework underpinning our own use of ELA and ROI-based connectivity mapping. For example, Ghazi et al. (2025) recently leveraged advanced fMRI-based connectomics to identify brain network markers that predict response to transcranial magnetic stimulation in cocaine use disorder (Ghazi et al., 2025), further reinforcing the utility and precision of fMRI-derived network measures in clinical settings. By situating our current findings within this broader tradition, the present results gain added relevance: demonstrating that disrupted cerebellar–temporal–visual connectivity in cocaine users fits into a larger context of pharmacologically induced alterations in human brain networks, where methodological rigor and sensitivity to subtleties in network structure are paramount. Thus, while aspects of our findings do mirror broader trends in pharmacologically induced brain connectivity changes, the distinct features especially in the directionality and state-dependent properties of cerebellar network dysregulation suggest a degree of specificity to chronic cocaine exposure and its impact on dopamine-dependent neuroplasticity. This interpretation is supported by Ghazi et al.'s demonstration that connectivity markers can predict treatment response, implying that the observed patterns may serve as mechanistically relevant biomarkers, rather than simply reflecting a class effect of stimulants or general neuromodulation.

Nevertheless, several limitations should be acknowledged. First, this study's cross-sectional design prevents causal inference regarding whether altered connectivity is a predisposing factor or a consequence of cocaine use. Second, potential confounders such as polysubstance use and comorbid psychiatric conditions were not explicitly modeled. Third, while ELA offers a unique perspective on network stability, the number of regions and temporal resolution used in this analysis could influence state estimation and energy computation. Longitudinal, multimodal studies combining diffusion imaging and behavioral measures would provide stronger validation of these findings. Further, fMRI methodology is susceptible to motion and physiological artifacts, and while denoising and quality assurance steps were robust, residual confounds cannot be excluded.

In conclusion, our findings reveal a distinct pattern of cerebellar–visual–temporal dysconnectivity and disrupted dynamic stability in cocaine users. These alterations reflect reduced neural efficiency and flexibility; potentially underlying impaired cognitive and perceptual regulation associated with addiction. By elucidating the cerebellum's role in addiction-related network dysregulation, this study contributes to a more comprehensive understanding of the neurobiological mechanisms of stimulant use disorder and highlights potential targets for biomarker-driven therapeutic intervention.

## 5 Conclusion

This study advances our understanding of the altered functional organization and temporal dynamics of large-scale brain networks in cocaine users (CUs) through a data-driven Energy Landscape Analysis (ELA) framework. By integrating ROI-to-ROI connectivity mapping and ELA-derived state modeling, we identified distinct patterns of cerebellar, temporal, and visual network dysconnectivity that differentiate CUs from healthy controls (HCs). The antagonistic coupling between the cerebellum and the inferior temporal gyrus (toITG), together with disrupted vermis–visual interactions, suggests a breakdown in cerebellar-mediated modulation of sensory and associative processes. These alterations likely contribute to the impaired cognitive control, perceptual rigidity, and reward dysregulation characteristic of chronic cocaine use.

The temporal dynamics findings further reveal that HCs engage low-energy, stable connectivity states more frequently and transition among them more flexibly than CUs. This pattern indicates preserved network adaptability and efficient neural regulation in HCs, whereas CUs exhibit reduced access to these “regulatory” states, reflecting diminished network resilience. Such deficits may underlie persistent maladaptive behaviors and the diminished capacity for cognitive and emotional self-regulation observed in addiction.

Overall, the combined evidence highlights the cerebellum's underappreciated role in addiction-related network dysfunction and positions ELA as a powerful tool for probing nonlinear brain dynamics in substance use disorders. Future longitudinal and multimodal studies should determine whether these altered energy landscapes represent reversible adaptations to cocaine exposure or enduring vulnerability markers. By elucidating these dynamic network signatures, this work lays the foundation for developing targeted interventions—such as neuromodulation or cognitive rehabilitation—aimed at restoring network flexibility and enhancing recovery in cocaine addiction.

## CRediT authorship contribution statement

**Sravani Varanasi:** Writing – review & editing, Writing – original draft, Visualization, Validation, Software, Methodology, Investigation, Formal analysis, Conceptualization. **Tianye Zhai:** Writing – review & editing, Validation, Supervision, Data curation, Conceptualization. **Hong Gu:** Writing – review & editing, Validation, Supervision, Data curation, Conceptualization. **Betty Jo Salmeron:** Writing – review & editing, Validation, Supervision, Conceptualization. **Yihong Yang:** Writing – review & editing, Validation, Supervision, Conceptualization. **Fow-Sen Choa:** Writing – review & editing, Validation, Supervision, Resources, Project administration, Methodology, Funding acquisition, Data curation, Conceptualization.

## Funding sources

The authors Tianye Zhai, Hong Gu, Betty Jo Salmeron and Yihong Yang were supported by the 10.13039/100030692Intramural Research Program of 10.13039/100000026National Institute on Drug Abuse, National Institutes of Health.

## Declaration of competing interest

The authors have nothing to declare.

## Data Availability

The data supporting the findings of this study are available within the Article. Raw data that support the findings of this study are available upon request and approval from the corresponding author.
