# Supplementary material for: Resting-state fMRI analysis of functional connectivity and temporal dynamics differences between cocaine users and healthy controls
Source: Neuroimage Rep. 2025 Dec 17;6(1):100304. doi: 10.1016/j.ynirp.2025.100304 (PMC12771301; doi:10.1016/j.ynirp.2025.100304)
Supplement: Multimedia component 1 [file mmc1.docx]

The 132 ROIs and their corresponding network affiliation as per the CONN Harvard-Oxford atlas are shown in Table S1 below. The l,r in the ROI names in Table S1 indicate the left and right hemispheres of the brain.

| ROI name | Network affiliation |
| --- | --- |
| Cingulate Gyrus, posterior division | posterior Default Mode Network |
| Precuneous Cortex |  |
| Middle Temporal Gyrus, anterior division – l,r | anterior Default Mode Network |
| Frontal Medial Cortex |  |
| Subcallosal Cortex |  |
| Paracingulate Gyrus – l,r | Paracingulate Gyrus |
| Thalamus – l,r | Thalamus |
| Caudate – l,r |  |
| Accumbens – l,r |  |
| Frontal Pole Right | Fronto Parietal Network, right hemisphere |
| Superior Frontal Gyrus Right |  |
| Middle Frontal Gyrus Right |  |
| Inferior Frontal Gyrus, pars opercularis Right | Language Network, right Hemisphere |
| Middle Temporal Gyrus, temporooccipital part Right |  |
| Supramarginal Gyrus, posterior division Right |  |
| Parahippocampal Gyrus, anterior division – l,r | Limbic & anterior Para hippocampal Cortex |
| Temporal Fusiform Cortex, anterior division – l,r |  |
| Temporal Fusiform Cortex, posterior division – l,r |  |
| Hippocampus – l,r |  |
| Amygdala – l,r |  |
| Parahippocampal Gyrus, posterior division – l,r | Cerebellum & posterior Para hippocampal Cortex |
| Brain-Stem |  |
| Cerebelum 3 – l,r |  |
| Cerebelum 9 – l,r |  |
| Vermis 1 2 |  |
| Vermis 3 |  |
| Vermis 10 |  |
| Inferior Frontal Gyrus, pars triangularis Right | Inferior Temporal Gyrus |
| Inferior Temporal Gyrus, anterior division – l,r |  |
| Inferior Temporal Gyrus, posterior division – l,r |  |
| Frontal Orbital Cortex – l,r |  |
| Frontal Pole Left | Fronto Parietal & Language Network, left hemisphere |
| Superior Frontal Gyrus Left |  |
| Middle Frontal Gyrus Left |  |
| Inferior Frontal Gyrus, pars triangularis Left |  |
| Inferior Frontal Gyrus, pars opercularis Left |  |
| Middle Temporal Gyrus, temporooccipital part Left |  |
| Supramarginal Gyrus, posterior division Left |  |
| Precentral Gyrus – l,r | Motor areas |
| Postcentral Gyrus – l,r |  |
| Insular Cortex – l,r | Auditory areas |
| Juxtapositional Lobule Cortex -formerly Supplementary Motor Cortex – l,r |  |
| Central Opercular Cortex – l,r |  |
| Parietal Operculum Cortex – l,r |  |
| Planum Polare – l,r |  |
| Heschl's Gyrus – l,r |  |
| Planum Temporale – l,r |  |
| Lateral Occipital Cortex, superior division – l,r | superior Latereal Occipital Cortex |
| Cerebelum Crus1 – l,r | Cerebellar Crus |
| Cerebelum Crus2 – l,r |  |
| Cerebelum 7b – l,r |  |
| Middle Temporal Gyrus, posterior division – l,r | lateral Default Mode Network areas |
| Angular Gyrus – l,r |  |
| Temporal Pole – l,r | Superior Temporal Gyrus |
| Superior Temporal Gyrus, anterior division – l,r |  |
| Superior Temporal Gyrus, posterior division – l,r |  |
| Supramarginal Gyrus, anterior division – l,r | anterior Supramarginal Gyrus |
| Cingulate Gyrus, anterior division | Salience network |
| Frontal Operculum Cortex – l,r |  |
| Putamen – l,r |  |
| Pallidum – l,r |  |
| Intracalcarine Cortex – l,r | primary Visual areas |
| Cuneal Cortex – l,r |  |
| Lingual Gyrus – l,r |  |
| Supracalcarine Cortex – l,r |  |
| Lateral Occipital Cortex – l,r | secondary Visual areas |
| Temporal Occipital Fusiform Cortex – l,r |  |
| Occipital Fusiform Gyrus – l,r |  |
| Occipital Pole – l,r |  |
| Inferior Temporal Gyrus, temporooccipital – l,r | Dorsal Attention Network |
| Superior Parietal Lobule – l,r |  |
| Cerebelum 4 5 – l,r | Cerebellum |
| Cerebelum 6 – l,r |  |
| Cerebelum 8 – l,r |  |
| Cerebelum 10 – l,r |  |
| Vermis 4 5 |  |
| Vermis 6 |  |
| Vermis 7 |  |
| Vermis 8 |  |
| Vermis 9 |  |

Table S1: CONN Harvard-Oxford atlas ROI clusters.
